# 细胞因子组合辅助诊断眼部慢性移植物抗宿主病的临床研究

**DOI:** 10.3760/cma.j.cn121090-20231031-00242

**Published:** 2024-03

**Authors:** 先静 程, 锐 吉, 瑞昊 黄, 世勤 黄, 围 范, 元程 赵, 容娣 袁, 筱淇 王, 曦 张

**Affiliations:** 1 陆军军医大学第二附属医院血液病医学中心，全军临床重点专科，创伤与化学中毒国家重点实验室，重庆市医学重点学科，血液病与微环境重庆市重点实验室，重庆 400037 Medical Center of Hematology, Xinqiao Hospital of Army Medical University, Chongqing Key Laboratory of Hematology and Micoenvironment, State Key Laboatory of Trauma and Chemical Poisoning, Chongqing 400037, China; 2 陆军军医大学新桥医院眼科，重庆 400037 Department of Ophthalmology, Xinqiao Hospital, Army Medical University, Chongqing 400037, China; 3 金凤实验室，重庆 400037 Jinfeng Laboratory, Chongqing 400037, China

**Keywords:** 异基因造血干细胞移植, 眼部慢性移植物抗宿主病, 泪液, 细胞因子, 生物标志物, Allogeneic hematopoietic stem cell transplantation, Ocular cGVHD, Tear fluid, Cytokine, Biomarker

## Abstract

**目的:**

探索细胞因子与眼部慢性移植物抗宿主病（cGVHD）的相关性，筛选眼部cGVHD的特异性生物标志物。

**方法:**

通过建立小鼠cGVHD模型，探讨cGVHD与血清细胞因子相关性。根据动物实验结果和文献检索，确定了16种细胞因子组合，使用酶联免疫吸附试验（ELISA）比较从2017年6月至2022年3月在陆军军医大学新桥医院血液病医学中心接受异基因造血干细胞移植后发生眼部cGVHD和未发生眼部cGVHD患者的血清与泪液细胞因子表达水平。

**结果:**

①与对照组比较，cGVHD小鼠血清IL-1β、IL-6、IL-8、IL-17、IFN-γ、CX3CL1、CXCL11、CXCL13、CCL11、CCL19浓度升高（*P*<0.05）；②患者血清和泪液细胞因子检测中，与未发生眼部cGVHD患者相比，眼部cGVHD患者血清细胞因子IL-8浓度升高（*P*＝0.032，AUC＝0.678）、IL-10浓度降低（*P*＝0.030，AUC＝0.701），泪液IL-8、IFN-γ、CXCL9和CCL17表达水平升高，而IL-10和CCL19表达水平较低（*P*<0.05，AUC值均>0.7），并且泪液细胞因子与眼部cGVHD疾病眼表参数相关。

**结论:**

泪液细胞因子较血清细胞因子诊断眼部cGVHD更具有特异性与敏感性。泪液细胞因子IL-8、IL-10、IFN-γ、CXCL9、CCL17和CCL19可以作为移植后眼部cGVHD诊断性生物标志物，对其临床诊断具有一定价值。

## Abstract (en)

**Objective:**

To investigate the association between cytokines and ocular chronic graft-versus-host disease（cGVHD）and identify specific biomarkers for ocular cGVHD to enhance clinical diagnosis, treatment, and evaluation.

**Methods:**

A mouse model of cGVHD was established to explore the correlation between cGVHD and serum cytokines. Based on the findings from the animal experiments and literature review, a panel of 16 cytokine combinations was identified. Enzyme-linked immunosorbent assay（ELISA）was used to compare the cytokine concentrations in the serum and tear samples from patients who underwent allogeneic hematopoietic stem cell transplantation from June 2017 to March 2022 at the Medical Center of Hematology, Xinqiao Hospital, Army Medical University.

**Results:**

① Compared with the control group, mice with cGVHD exhibited elevated serum IL-1β, IL-6, IL-8, IL-17, IFN-γ, CX3CL1, CXCL11, CXCL13, CCL11, and CCL19 concentrations（all *P*<0.05）. ② Analysis of the cytokine profiles of the serum and tear samples revealed that compared with patients without ocular cGVHD, those with ocular cGVHD exhibited increased serum IL-8［*P*＝0.032, area under the curve（AUC）＝0.678］; decreased serum IL-10（*P*＝0.030, AUC＝0.701）; elevated IL-8, IFN-γ, CXCL9, and CCL17 in tear samples; and lower IL-10 and CCL19 in tear samples（all *P*<0.05, all AUC>0.7）. Moreover, cytokines in tear samples showed correlations with ocular surface parameters related to ocular cGVHD.

**Conclusion:**

Tear fluid demonstrates greater specificity and sensitivity as a biomarker for diagnosing ocular cGVHD than serum biomarkers. Among the identified cytokines in tear samples, IL-8, IL-10, IFN-γ, CXCL9, CCL17, and CCL19 serve as diagnostic biomarkers for ocular cGVHD post-transplantation, offering practical reference value for diagnosis.

慢性移植物抗宿主病（cGVHD）是异基因造血干细胞移植（allo-HSCT）的主要远期并发症及死亡原因，发生率为30％～70％[1]–[2]。眼部cGVHD的发生率为40％～60％，常表现为干眼、视物模糊、视力下降甚至丧失，严重影响患者生存质量[3]。目前，眼部cGVHD诊断主要依据眼部症状及眼科检查，缺乏特异性指标，且有创检查会破坏患者眼表结构，不利于长期随访[4]–[5]。因此，临床亟需安全方便、灵敏有效的眼部cGVHD诊断方式。

生物标志物检测简单安全、快速有效，有利于疾病早期筛查和诊断。此前，本研究团队前期报道TLR4、TNFR1、TGF-β和Elafin可作为生物标志物组合，辅助临床急性GVHD患者诊断、分级和对激素治疗敏感性评估，对临床应用具有重大意义[6]。在cGVHD生物标志物研究中，我们发现CXCL9、CXCL13、CCL11、CCL17和CCL23五种细胞因子可以作为早期诊断、评估cGVHD发生的特异性生物标志物组合。其中，CXCL9和CCL17还可为肝脏、皮肤靶器官损害提供靶向参考[7]。此外，部分研究报道泪液细胞因子有望成为眼部cGVHD诊断、治疗与监测预后的生物标志物[8]。但由于发现的细胞因子数量较少，且不同研究发现的特定细胞因子之间存在差异，尚缺乏可作为眼部cGVHD诊断金标准的公认细胞因子。国内外也缺乏比较血清与泪液生物标志物诊断眼部cGVHD的相关研究。

本研究通过构建cGVHD小鼠模型，使用酶联免疫吸附试验（ELISA）检测cGVHD动物血清细胞因子，确定下一步临床所需检测的细胞因子组合，并用于测定allo-HSCT后cGVHD患者血清和泪液细胞因子的表达水平，以期筛选具有辅助诊断眼部cGVHD的特异性生物标志物，为临床诊断提供参考。

## 对象与方法

一、实验动物

SPF级BALB/C，8～12周龄，雄性，购买于北京斯贝特生物技术有限公司，SPF级B10.D2（JAX:000461），8～12周龄，雄性，购买于The Jackson Laboratory，并寄养于维通利华实验动物技术有限公司。动物实验获得陆军军医大学实验动物福利伦理审查委员会审核，符合伦理和动物福利要求（AMUWEC20211606）。

二、cGVHD小鼠模型构建

将BALB/c小鼠随机装于无菌辐照盒内，按要求放于辐照台上，给予7.5 Gy（γ射线）的剂量全身辐照，8 h后对小鼠行尾静脉注射。注射前按照1∶1的方式区组随机化对受鼠BALB/c进行分组且每只受鼠的尾静脉注射液保持200 µl。cGVHD组输注B10.D2供鼠来源骨髓细胞（10×10^6^/只）和脾脏细胞（18×10^6^/只），BM组（对照组）输注骨髓细胞（10×10^6^/只）。移植结束后将受鼠送回SPF级动物房，观察并分析两组小鼠体重变化、生存状态与生存率、靶器官病理组织切片，明确组织受累程度。

三、病例

本研究纳入2017年6月至2022年3月在陆军军医大学新桥医院血液病医学中心接受allo-HSCT的45例患者，其中26例发生眼部cGVHD（眼部cGVHD组），19例未发生眼部cGVHD（对照组）。眼部cGVHD组纳入标准：临床症状与眼科专科医师检查诊断眼部cGVHD且无其他急性眼部疾病与其他慢性眼部疾病、近3个月内未做过眼科手术。对照组纳入标准：移植后发生cGVHD但未累及眼部、无慢性或者活动性眼部感染、近3个月内未做过眼科手术。参考2014年NIH慢性GVHD全局评分法对所有受试者进行疾病严重程度分级，可分为轻度、中度、重度[2],[9]。眼部cGVHD组与对照组患者一般资料与移植特征详见表1，眼表参数情况详见表2。临床研究获得陆军军医大学新桥医院伦理委员会批准（批件号：2022-研第215-01）。

**表1:** 眼部慢性移植物抗宿主病（cGVHD）组与对照组的一般资料与移植特征

基本特征	眼部cGVHD组（26例）	对照组（19例）
年龄［岁，*M*（范围）］	38（19~61）	30（18~56）
性别［例（%）］		
男	14（53.8）	9（47.4）
女	12（46.2）	10（52.6）
疾病类型［例（%）］		
ALL	4（15.4）	5（26.3）
AML	10（38.5）	10（52.6）
CML	3（11.5）	1（5.3）
MDS	6（23.1）	2（10.5）
AA	0（0.0）	1（5.3）
其他	3（11.5）	0（0.0）
供患者类型［例（%）］		
有血缘	20（76.9）	14（73.7）
其他	6（23.1）	5（26.3）
干细胞来源［例（%）］		
PBSC	21（80.8）	14（73.7）
PB+BM	5（19.2）	5（26.3）
器官受累［例（%）］		
皮肤	9（34.6）	8（42.1）
肝脏	6（23.1）	10（52.6）
口腔	4（15.4）	2（10.5）
肺部	4（15.4）	3（15.8）
胃肠道	0（0.0）	0（0.0）
关节	0（0.0）	1（5.3）
生殖器	0（0.0）	0（0.0）
cGVHD严重程度［例（%）］		
轻度	6（23.1）	6（31.6）
中度	12（46.2）	8（42.1）
重度	8（30.7）	5（26.3）

注 ALL：急性淋巴细胞白血病；AML：急性髓系白血病；CML：慢性髓性白血病；MDS：骨髓增生异常综合征；AA：再生障碍性贫血；PBSC：外周血造血干细胞；BM：骨髓

**表2:** 眼部慢性移植物抗宿主病（cGVHD）组与对照组眼表参数比较

眼表参数	眼部cGVHD组（26例）	对照组（19例）	*P*值
OSDI评分	50.06±20.70	9.87±9.47	<0.001
Schirmer Ⅰ试验	3.92±3.33	22.58±8.30	<0.001
FTBUT	3.46±2.44	10.79±2.64	<0.001
CFS评分	9.73±5.33	0.42±0.77	<0.001
NIH评分（眼）	1.73±0.72	0	<0.001

注 OSDI：眼表疾病指数；Schirmer Ⅰ：无麻醉的Schirmer试验；FTBUT：荧光素染色泪液破裂时间；CFS：角膜荧光素染色

四、细胞因子检测

泪液与血清标本采集时间点：眼部cGVHD组发生眼部cGVHD但未进行眼部药物干预时（除人工泪液外），对照组发生cGVHD但未进行全身药物干预时。应用中国江苏酶免实业有限公司血清/泪液蛋白标志物试剂盒，采用ELISA检测以下16种细胞因子：IL-1β、IL-6、IL-8、IL-10、IL-17、CX3CL1、CXCL9、CXCL11、CXCL13、CCL11、CCL17、CCL19、CCL21、CCL23、IFN-γ和BAFF。

五、统计学处理

使用SPSS 26.0统计软件分析统计相关数据。使用Shapiro-Wilk检验方法验证正态性假设。正态分布 的计量资料，采用*t*检验进行分析，以“平均值±标准差”表示；Mann-Whitney *U*检验分析非正态分布数据。使用卡方检验比较性别等定性数据。使用Kaplan-Meier曲线进行小鼠生存分析，受试工作特征曲线（ROC）分析细胞因子诊断能力，Spearman检验用于分析相关性。

## 结果

一、cGVHD小鼠模型的建立和鉴定

观察小鼠体重变化及生存状态，并进行相关cGVHD动物表现评分[10]。体重结果显示，BM组小鼠移植初期体重减轻，随时间进展体重逐渐恢复到基线水平；cGVHD组小鼠体重随移植天数逐渐下降并伴有cGVHD评分增高（*P*<0.05）；生存分析显示，BM组小鼠全部存活，而cGVHD组从移植后第30 d开始症状逐渐加重，出现死亡（*P*<0.05）；cGVHD评分结果显示，cGVHD小鼠逐渐出现皮肤全身及眼部脱毛、眼睑水肿、皮肤瘢痕明显等典型的cGVHD表现。移植后45 d取受鼠泪腺进行组织切片病理观察。经HE染色后，可见泪腺腺体萎缩，局部散在浸润的淋巴细胞（图1）。

**图1:**
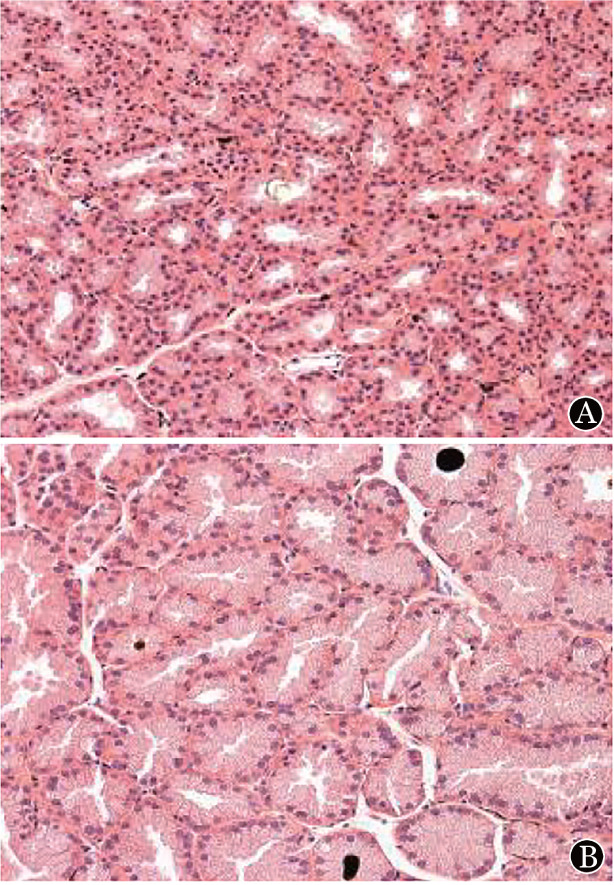
慢性移植物抗宿主病小鼠（A）与BM组（对照组）小鼠（B）移植后45 d泪腺组织变化

二、血清细胞因子与小鼠cGVHD相关性分析

细胞因子检测结果分析显示：cGVHD小鼠血清细胞因子IL-1β、IL-6、IL-8、IL-17、IFN-γ、CX3CL1、CXCL11、CXCL13、CCL11、CCL19表达高于对照组（*P*<0.05），其他血清细胞因子表达差异无统计学意义（表3）。

**表3:** 眼部慢性移植物抗宿主病（cGVHD）小鼠和对照组小鼠血清细胞因子水平（µg/L，*x±s*）

细胞因子	慢性cGVHD组（5只）	对照组（5只）	*P*值
IL-1β	120.05±14.01	93.69±20.61	0.014
IL-6	78.85±7.46	55.11±8.92	<0.001
IL-8	221.67±30.16	181.12±29.13	0.023
IL-10	31.60±6.82	40.50±9.23	0.059
IL-17	40.45±6.04	28.29±7.55	0.005
IFN-γ	582.45±56.95	413.47±47.19	<0.001
CX3CL1	809.43±62.86	695.80±77.22	0.012
CXCL9	953.77±157.28	814.50±101.50	0.115
CXCL11	1 521.29±226.60	1 142.87±182.67	0.006
CXCL13	308.65±32.42	238.69±54.16	0.011
CCL11	1 713.33±262.71	1 256.15±195.47	0.005
CCL17	883.55±143.96	748.14±153.68	0.111
CCL19	892.01±120.27	651.76±93.28	0.001
CCL21	677.88±144.01	526.85±135.17	0.074
CCL23	984.87±144.79	799.93±174.86	0.069
BAFF	1 306.80±222.99	1 073.46 ±194.20	0.077

注 IFN-γ：γ干扰素；CXCL：趋化因子CXC受体；CCL：趋化因子CC受体；CX3CL：趋化因子CX3C受体；BAFF：B细胞激活因子

三、眼部cGVHD患者血清与泪液细胞因子检测结果

1. 眼部cGVHD组与对照组患者血清细胞因子比较：与对照组相比，眼部cGVHD患者血清细胞因子IL-8表达水平增加（*P*＝0.032）而IL-10水平降低（*P*＝0.031），具体见表4。为了进一步验证血清细胞因子IL-8和IL-10诊断眼部cGVHD的潜力，我们进行了ROC分析，结果显示：IL-8 曲线下面积（AUC）＝0.678，IL-10 AUC＝0.701（图2）。此外，眼表参数是评估眼部疾病的严重程度的重要因素。我们使用Spearman检验方法分析血清细胞因子IL-8、IL-10与OSDI评分、眼部NIH评分、Schirmer试验、FTBUT与CFS评分等眼表参数之间的相关性，但未观察到血清细胞因子IL-8、IL-10与各眼表参数的关系有统计学意义（*P*>0.05）。

**表4:** 眼部慢性移植物抗宿主病（cGVHD）组和对照组患者血清细胞因子水平（µg/L，*x±s*）

细胞因子	眼部cGVHD组（22例）	对照组（19例）	*P*值
IL-1β	41.98±3.38	42.19±3.25	0.842
IL-6	12.54±1.31	13.18±1.37	0.133
IL-8	1 308.76±91.06	1 240.96±82.21	0.032
IL-10	266.24±31.88	298.84±32.48	0.030
IL-17	19.08±2.04	19.19±1.54	0.860
IFN-γ	484.36±38.34	486.48±29.97	0.847
CX3CL1	3.71±0.42	3.73±0.41	0.836
CXCL9	719.09±63.02	717.96±60.70	0.954
CXCL11	471.96±50.96	454.15±38.06	0.218
CXCL13	93.71±8.38	92.21±7.26	0.547
CCL11	299.88±24.15	311.49±23.78	0.130
CCL17	450.40±33.86	453.07±34.53	0.804
CCL19	212.96±16.88	207.74±25.09	0.433
CCL21	128.65±10.93	126.92±8.68	0.582
CCL23	135.81±9.60	139.88±11.22	0.218
BAFF	37.75±3.38	38.84±3.83	0.339

注 IFN-γ：γ干扰素；CXCL：趋化因子CXC受体；CCL：趋化因子CC受体；CX3CL：趋化因子CX3C受体；BAFF：B细胞激活因子

**图2:**
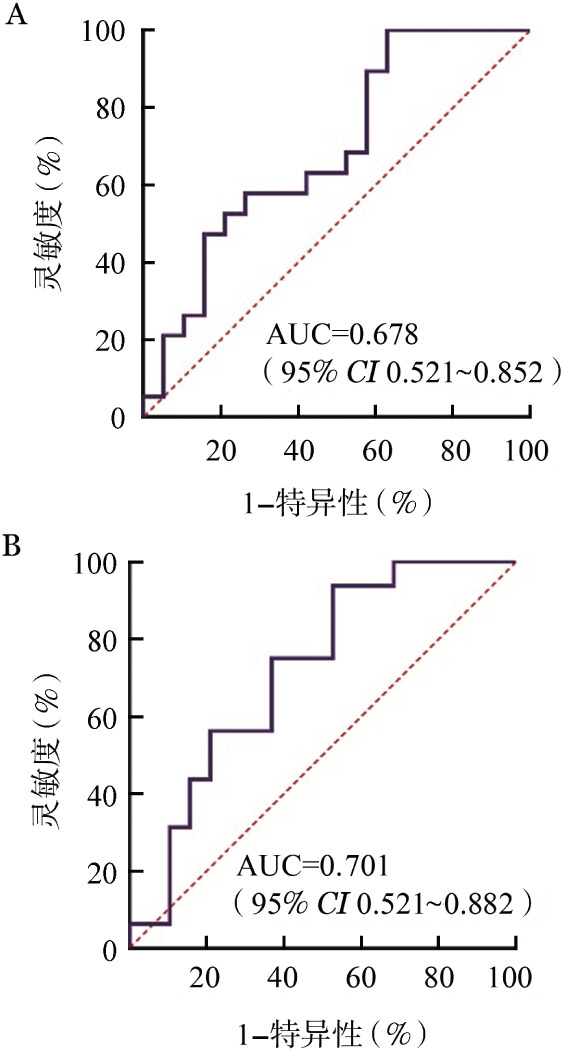
受试者工作特征曲线分析血清IL-8（A）、IL-10（B）诊断眼部慢性移植物抗宿主病（cGVHD）能力

2. 眼部cGVHD组与对照组患者泪液细胞因子比较：眼部cGVHD患者泪液细胞因子IL-8、IFN-γ、CXCL9和CCL17表达水平较对照组升高（*P*<0.05），IL-10和CCL19水平降低（*P*<0.05），具体详见表5。ROC曲线分析结果显示：所有泪液细胞因子AUC均>0.7，其中IL-10与CCL17的AUC>0.8。Spearman检验结果分析显示，细胞因子IL-10、IFN-γ和CXCL9与眼表参数相关，具有统计学意义（*P*<0.05）。其中，泪液IL-10水平与NIH评分呈负相关（*r*＝−0.431，*P*＝0.026），泪液IFN-γ水平与FTBUT呈负相关（*r*＝−0.526，*P*＝0.006），泪液CXCL9水平与CFS评分呈正相关（*r*＝0.480，*P*＝0.013）。

**表5:** 眼部慢性移植物抗宿主病（cGVHD）组和对照组泪液细胞因子水平（µg/L，*x±s*）

细胞因子	眼部cGVHD组（26例）	对照组（14例）	*P* 值
IL-1β	40.16±5.45	38.05±5.05	0.251
IL-6	22.20±2.96	22.37±3.63	0.092
IL-8	1 103.13±121.74	935.66±202.75	0.004
IL-10	408.62±16.05	431.88±8.53	0.001
IL-17	21.34±3.69	20.00±4.08	0.299
IFN-γ	432.17±42.12	392.51±43.44	0.010
CX3CL1	3.89±0.27	3.87±0.20	0.768
CXCL9	807.67±189.18	645. 57±96.85	0.012
CXCL11	573.98±69.98	552.38±37.97	0.256
CXCL13	81.98±4.83	78.40±10.75	0.160
CCL11	407.21±184.75	349.37±157.38	0.340
CCL17	386.09±37.59	339.54±37.24	0.004
CCL19	228.06±19.12	252.49±33.32	0.012
CCL21	120.29±20.82	109.30±22.77	0.132
CCL23	123.27±23.96	113.17±19.10	0.182
BAFF	44.84±6.14	47.10±5.79	0.265

注 IFN-γ：γ干扰素；CXCL：趋化因子CXC受体；CCL：趋化因子CC受体；CX3CL：趋化因子CX3C受体；BAFF：B细胞激活因子

## 讨论

眼部cGVHD是由免疫介导的眼表疾病，以慢性炎症和纤维化为主要特征[11]。主要是由T细胞浸润，引发免疫细胞激活与募集、炎性细胞因子和趋化因子的产生等一系列免疫炎症级联反应[12]–[13]。由此可见，细胞因子在眼部cGVHD病理生理过程的潜在机制中起着至关重要的作用。缺乏可靠和特异的早期临床指标，是眼部cGVHD早期临床治疗的一大阻碍，常导致延迟治疗与预后不良。通过研究眼部cGVHD生物标志物可有助于临床安全简便、快速有效的诊断眼部cGVHD。

本研究结果显示眼部cGVHD患者血清IL-8水平增加、IL-10水平降低，泪液IL-8、IFN-γ、CXCL9和CCL17水平升高、IL-10和CCL19水平降低，表明血清IL-8、IL-10和泪液IL-8、IL-10、IFN-γ、CXCL9、CCL17、CCL19均可能参与眼部cGVHD的发生发展，血清与泪液IL-8和IL-10在眼部cGVHD患者检测中变化趋势相同，说明血清细胞因子与泪液细胞因子在眼部cGVHD诊断中具有一定的一致性。但通过ROC分析显示，泪液生物标志物较血清生物标志物诊断眼部cGVHD更具有特异性与灵敏度。此外，眼表参数是临床上用于眼部疾病严重程度评估的重要手段。通过分析入组患者眼表指标，我们观察到眼部cGVHD患者OSDI、CFS和NIH眼部评分显著增加，Schirmer试验和FTBUT降低，这表明移植后cGVHD中发生眼部受累的患者泪液分泌减少、泪膜稳定性降低、角膜受损，整体眼表情况变差，与此前研究结果一致[14]。通过Spearman检验分析法，发现眼部cGVHD患者泪液细胞因子IL-10、IFN-γ和CXCL9与其眼表参数相关，这表明泪液细胞因子可反应眼部cGVHD严重程度。

IL-8对T淋巴细胞和中性粒细胞具有较强趋化性，可参与泪腺功能减退和眼表受损[15]。本研究发现眼部cGVHD患者泪液IL-8升高，与此前研究结果一致[16]–[18]。IL-8通路还被报道与许多炎症/瘢痕性的眼部疾病有关，提示可能参与眼部cGVHD瘢痕样改变[19]。本研究也发现血清和泪液IL-10均降低。但据报道，血清IL-10浓度变化在cGVHD临床研究中变化趋势不一致[20]–[21]。我们发现眼部cGVHD泪液IL-10浓度降低，与Serapicos等[18]研究结果一致，但与Nair等[22]研究结果不一致。该差异性可能与IL-10在GVHD中的双向调节功能有关，涉及到免疫调节和免疫刺激[23]–[25]。T细胞免疫失调在cGVHD慢性炎症反应阶段起重要作用[26]，而IFN-γ为辅助性T细胞（Th）1的标志性细胞因子。据报道，IFN-γ在cGVHD中具有双重作用，可增加趋化因子受体表达来诱导cGVHD，也可触发激活供体T细胞凋亡来抵御cGVHD[27]。我们发现眼部cGVHD组患者泪液IFN-γ升高，与多项研究结果一致[22],[28]。研究报道，IFN-γ表达增加可促使干眼患者结膜杯状细胞丢失与黏蛋白缺乏，为后续评估和诊治眼部cGVHD结膜损伤提供依据[29]。通过研究IFN-γ在眼部cGVHD的表达水平，有助于我们进一步探索眼部cGVHD免疫机制。

眼部cGVHD组织相关免疫途径被激活后，多种细胞因子和趋化因子上调，导致组织损伤破坏[30]。但目前趋化因子与眼部cGVHD关系的研究较少，尚不清楚趋化因子在眼部cGVHD的作用。本研究发现眼部cGVHD患者泪液CXCL9和CCL17水平升高，而CCL19水平降低。Westekenper等[31]研究也发现眼部cGVHD患者结膜CXCL9表达升高。CXCL9可以通过与其受体CXCR3结合，驱动供体效应T细胞（特别Th1细胞）进入cGVHD靶器官[32]。此外，CXCR3途径和IFN-γ之间的相互作用会在cGVHD中导致炎症反应加重[33]。CCL17也称为胸腺活化调节趋化因子，可以通过与CCR4（趋化因子受体4）结合发挥其生物学效应[34]。研究发现cGVHD患者血清CCL17增加，可诱导记忆T细胞的产生和淋巴细胞向炎症目标的迁移，同时破坏Th1和Th2平衡，促进cGVHD的发展[35]–[36]。CCL19作为胸腺和淋巴结中大量表达的一种稳态趋化因子，通过与其受体（CCR7）相互作用，可以协调淋巴器官组织、免疫应答启动和免疫耐受诱导。有研究表明CCL19可作为多种肿瘤的诊断和预后生物标志物，具有双重作用，包括调节免疫细胞转运诱导炎症以及诱导抗肿瘤效应[37]–[38]。

本研究存在一些局限性：①部分眼部cGVHD患者干眼严重，使用Schirmer试纸收集泪液较困难，影响结果判断。②为了进一步研究这些诊断细胞因子的准确性与可靠性，还需要进行大规模样本研究来验证结果等。③眼部cGVHD病理机制也尚未明确，还需构建与鉴别以眼部受累为主的cGVHD动物模型研究，进一步研究眼部cGVHD发病机制与新药开发。

综上，眼部cGVHD患者血清和泪液中均检测到与眼部cGVHD相关的细胞因子，但是泪液生物标志物较血清生物标志物诊断眼部cGVHD更特异与敏感。其中，泪液细胞因子IL-8、IL-10、IFN-γ、CXCL9、CCL17和CCL19可以作为眼部cGVHD的诊断性生物标志，对临床辅助诊断眼部cGVHD具有参考意义。
